# Hyperkalemia in a patient with myasthenia gravis: case presentation

**DOI:** 10.1186/s12902-019-0406-z

**Published:** 2019-07-26

**Authors:** Mi-Chu Lin, Ming-Hsien Tsai, Jyh-Gang Leu, Yu-Wei Fang

**Affiliations:** 1 Division of Nephrology, Department of Internal Medicine, Shin Kong Wu Ho-Su Memorial Hospital, Taipei, Taiwan, Republic of China; 2 Fu-Jen Catholic University School of Medicine, Taipei, Taiwan, Republic of China

**Keywords:** Autoimmune, Myasthenia gravis, Addison’s disease, Hyperkalemia, Transtubular potassium gradient

## Abstract

**Background:**

Myasthenia gravis (MG) is the most common disorder of neuromuscular transmission, and it is typified by fluctuating degrees and variable combinations of weakness in the ocular, bulbar, limb, and respiratory muscles. Under rare circumstances, MG can be accompanied by Addison’s disease.

**Case presentation:**

Here, we reported the case of a 57-year-old Chinese woman with MG. She experienced progressive muscle weakness for 1 week. MG with acute exacerbation was initially suspected. However, further biochemistry tests found mild hyperkalemia (5.6 mEq/L) and a lower renal potassium excretion rate. Consequently, low aldosterone action was highly suspected. Further findings included a suppressed cortisol level, a higher adrenocorticotropic hormone concentration, and 21-hydroxylase antibody positivity, supporting a diagnosis of primary adrenal insufficiency due to autoimmune adrenalitis.

**Conclusion:**

We successfully demonstrated that adrenal insufficiency could be diagnosed, due to the presence of hyperkalemia. This case suggested a need for clinicians to consider the possible coincidence of adrenal insufficiency in a patient with MG and hyperkalemia. Early hormone supplementation should be begun.

## Background

Myasthenia gravis (MG), an autoimmune disease caused by the blockade of nicotinic acetylcholine receptors in the junctions between the nerve and muscle by antibodies, is the most common disorder of neuromuscular transmission. More than 50% of MG patients present with ptosis and diplopia, and approximately 15% present with bulbar symptoms, including fatigable chewing, dysphagia, and dysarthria [1]. MG has well-known links with various autoimmune diseases, such as Hashimoto’s disease, Graves’ disease, rheumatoid arthritis, systemic lupus erythematosus, and type 1 diabetes mellitus [2, 3]. However, case reports of coexistent MG and Addison’s disease are rare.

In this study, we report a rare case of MG in a patient who presented with mild hyperkalemia, which finally led to a diagnosis of MG with primary adrenal insufficiency. We succeeded in diagnosing primary adrenal insufficiency early by considering mild hyperkalemia, which is easily neglected in clinical practice.

## Case presentation

A 57-year-old Chinese woman who was a carrier of the hepatitis B and C viruses and who had a 2-year history of MG was receiving treatment with oral Mestinon (pyridostigmine 120 mg daily), which controlled her symptoms well (minimal bilateral ptosis, especially in the morning, and proximal muscle power of 4/5). The diagnosis of MG was verified by the presence of high levels of immunoglobulin G antibodies directed against acetylcholine receptors (54.23 nmol/L). The stimulation single fiber electromyography recorded from orbicularis oculi muscle showed abnormal results (MCD: 180 s), and repetitive nerve stimulation showed no decremental response. A chest computed-tomography (CT) scan showed a thymoma of about 3.2 cm.

She presented with a 7-day history of poor appetite and progressive muscular weakness. Acute exacerbation of MG was initially considered by the neurologist but the muscle weakness did not improve in spite of an increased Mestinon dose to 360 mg daily. She was referred to the nephrology department for further assessment because the laboratory data showed an electrolyte imbalance. Such symptoms such as nausea, vomiting, diarrhea, tarry stool, and lower extremity numbness were absent. There was no history of recent strenuous exercise or diuretic use.

On admission, physical examination revealed clear consciousness and symmetrical weakness of the lower extremities. The following laboratory findings were recorded: blood pressure, 116/64 mmHg; heart rate, 82 beats/min; respiratory rate, 22 breaths/min; and body temperature, 36.8 °C. Moreover, a generalized skin pigmentation without long-term exposure to sunlight was noted. The remainder of the physical examination was unremarkable.

**Fig. 1:**
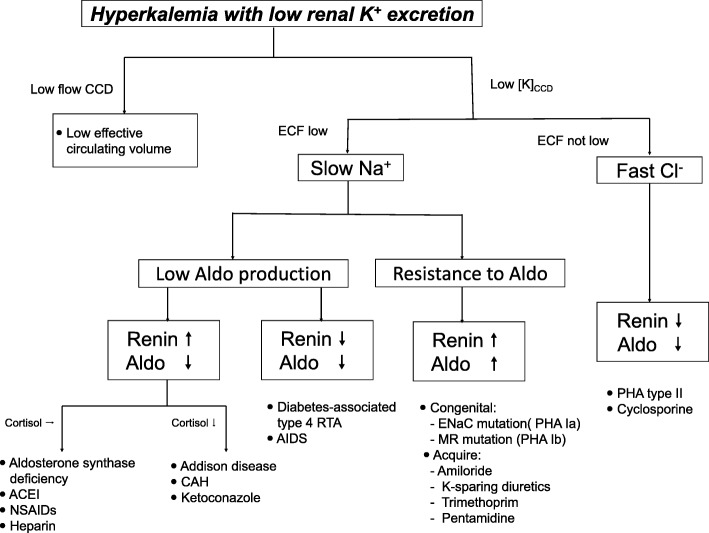
Schematic illustration of the recommended diagnostic approach to hyperkalemia with low renal potassium secretion rate [4–6]. Abbreviations: *CCD* cortical collecting duct, *ECF* extracellular fluid, *Aldo* aldosterone, *CAH* congenital adrenal hyperplasia, *ENaC* epithelial sodium channel, *NSAID* nonsteroidal anti-inflammatory drugs, *PHA* pseudohypoaldosteronism, *MR* mineralocorticoid receptor

**Fig. 2:**
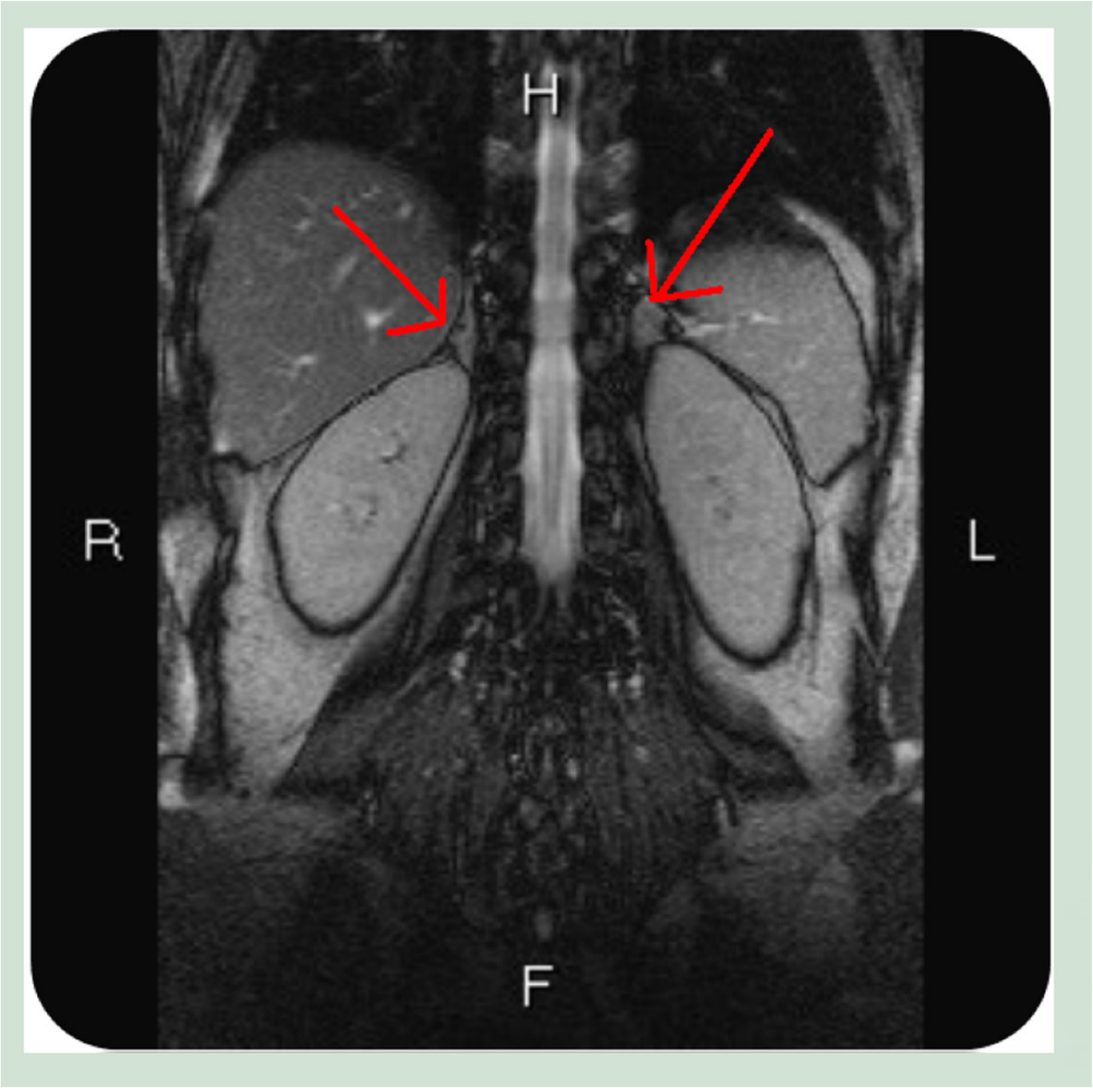
Abdominal magnetic resonance imaging revealed the relatively small size of the bilateral adrenal gland (arrow)

The results of the biochemical analyses are shown in Table 1. The most prominent initial findings were mild hyperkalemia (5.6 mEq/L) and mild hyponatremia (128 mEq/L). Serial urinalysis during admission indicated a decreased K^+^ excretion rate (transtubular potassium gradient [TTKG] = 2.64; urinary K^+^–creatinine ratio = 6.4 mmol/mmol). Based on the findings of hyperkalemia with low urine potassium secretion, low aldosterone action was highly suspected. Subsequent hormone profiling revealed normal thyroid-stimulating hormone levels (2.99 μg/dL), an elevated renin concentration (74.5 pg/mL), and low aldosterone (22.24 pg/mL), and cortisol levels (4.65 μg/dL) (Table 1). Accordingly, adrenal insufficiency was diagnosed using the flow chart of hyperkalemia differentiation (Fig. 1). Moreover, the patient’s elevated adrenocorticotropic hormone level (84.4 pg/mL) supported the diagnosis of primary adrenal insufficiency. Magnetic resonance imaging revealed bilateral shrinkage of the adrenal glands, confirming our diagnosis (Fig. 2). Because of the underlying disease of MG, autoimmune adrenalitis was highly suspected, and this was proven by 21-hydroxylase antibody positivity. Thereafter, the final diagnosis was primary adrenal insufficiency due to autoimmune adrenalitis.

**Table 1:** Laboratory data at admission

Parameters (reference range)	Value
Plasma	
PH (7.35–7.45)	7.40
Blood nitrogen (8–20 mg/dL)	13
Creatinine (0.5–1.5 mg/dL)	0.7
Na^+^ (133–145 mEq/L)	128^a^
K^+^ (3.3–5.1 mEq/L)	5.6^a^
Cl^−^ (96–108 mEq/L)	97
Calcium (8.4–10.2 mg/dL)	9.4
Phosphate (2.5–4.5 mg/dL)	3.7
Magnesium (1.5–2.5 mg/dL)	2.2
Osmolality (278–305 mOsm/kg. H2O)	265
Cortisol (6.2–19.4 μg/dL)	4.65
ACTH (5–77 pg/mL)	84.4
Renin (15–57 pg/mL)	74.5
Aldosterone (78–104 pg/mL)	22.24
iPTH (15–65 pg/mL)	66.79
TSH (0.35–4.94 μg/dL)	2.99
ANA	80+ homogeneous
Anti-ds DNA	20 × +
Spot urine	
K^+^ (mEq/L)	12
Na^+^ (mEq/L)	56
Cl^−^ (mEq/L)	44
Creatinine (μg/dl)	21.1
Osmolality (mOsm/kg H_2_O)	215
TTKG (<9^b^)	2.64
K^+^/Cr (mmol/mmol) (<15^b^)	6.4

Abbreviations: *ACTH* Adrenocorticotropic hormone, *TSH* thyrotropin, *ANA* anti-nuclear antibody, *TTKG* transtubular potassium gradient

^a^Indicates abnormal values

^b^Indicates reference range for normal renal response to hyperkalemia

Fludrocortisone (0.2 mg daily) was prescribed as mineralocorticoid supplementation. The patient’s serum electrolyte levels normalized, and her general weakness improved. Therefore, she was discharged with oral fludrocortisone (0.2 mg daily) treatment. At the 2-month follow-up, her serum K^+^ concentration was 3.68 mEq/L.

## Discussion

Primary adrenocortical insufficiency (also termed Addison’s disease) is caused by an impaired ability of the adrenal cortex to secrete glucocorticoids and mineralocorticoids, and secondary adrenal insufficiency is caused by hypothalamic–pituitary axis suppression. Adrenocortical insufficiency must be presumptively considered in the presence of a broad spectrum of patient symptoms (weight loss, abdominal pain, nausea, vomiting, cutaneous hyperpigmentation), changes in mood and personality, past medical history (diabetes, thyroid disease), and laboratory test data (hyperkalemia, hyponatremia, hypoglycemia, metabolic acidosis). If undetected or left untreated, adrenocortical insufficiency is associated with significant morbidity and mortality. Acute adrenal insufficiency is the result of critical life-threatening glucocorticoid and mineralocorticoid levels [7].

MG is a rare autoimmune disease caused by autoantibodies against the nicotinic acetylcholine receptor, the muscle specific tyrosine kinase or the low-density lipoprotein receptor-related protein 4 [8, 9]. A thymoma is found in 15% of all MG patients. Thymoma–MG may be associated with neuromyotonia, Sjogren’s syndrome, or autoimmune hemolytic anemia [10]. MG is accompanied by increased risk for other autoimmune disorders [11].

Polyglandular autoimmune syndrome (PAS) includes a wide spectrum of autoimmune disorders. PAS type 1, also termed autoimmune polyendocrinopathy-candidiasis-ectodermal dystrophy syndrome, is a rare autosomal recessive disorder of childhood [12]. Conversely, PAS type 2, which is far more prevalent than PAS type 1, occurs in adulthood. Primary adrenal insufficiency is the principal presentation of PAS type 2, and it is through autoimmune thyroid diseases (e.g., Hashimoto thyroiditis, Grave’s disease) or type 1 diabetes mellitus [13]. In rare cases, PAS type 2 includes MG [14–16]. A coincidence of MG and Addison’s disease has been reported [4–6, 17]. However, the present patient only presented with primary adrenal insufficiency and MG, indicating that a diagnosis of PAS type 2 is improper (Table 2).

**Table 2:** Patients with association of myasthenia gravis and Addison’s disease

No	Studies	Year	Sex	Age at MG onset (year)	Age at AD onset (year)	Symptom	Sodium (mEq/L)	Potassium (mEq/L)	PAS type 2
1.	Bosh et al. [14]	1977	Female	16	30	Fatigue, weakness	122	4.2	Yes (Hypothyroidism)
2	Dumas et al. [4]	1985	Female	48	35	Diplopia	NA	NA	No
3	Dumas et al. [4]	1985	Male	50	37	Diplopia	NA	NA	No
4	McAlpine et al. [15]	1988	Female	17	47	Nausea, vomiting	99	4.9	Yes (Hypothyroidism)
5	Kane et al. [5]	1950	Male	18	19	Nausea, vomiting	128	6.7	No
6	Okada et al. [6]	1994	Female	38	53	Nausea, diarrhea	113	3.8	No
7	Seker et al. [17]	2009	Male	32	32	Weakness, vomiting	144	3.8	No
8	Knno et al. [16]	2009	Female	74	61	Vomiting, headache	137	3.9	Yes (autoimmune thyroid disease)
9	Our case	2018	Female	55	57	Fatigue, weakness	128	5.6	No

Abbreviations: *MG* myasthenia gravis, *AD* Addison’s disease, *PAS* polyglandular autoimmune syndrome, *NA* not available

Our patient had no history of other autoimmune diseases even though she presented with mild hyperkalemia (5.6 mEq/L) and mild hyponatremia (128 mEq/L). Physicians may consider these symptoms insignificant. However, we successfully diagnosed primary adrenal insufficiency due to immune adenitis early based on the presence of mild hyperkalemia, whereas in other studies, diagnoses of the condition have occurred at later stages, as indicated by the presence of severe hyponatremia (Table 2).

The three primary causes of hyperkalemia are increased K^+^ intake, K^+^ release from cells, and impaired renal K^+^ excretion. In our case, TTKG was low under the hyperkalemic status, indicating impaired renal K^+^ excretion. A differential flow chart of hyperkalemia with low renal potassium secretion is shown in Fig. 2 [18–20]. First, impaired renal function and ineffective plasma volume should be eliminated as possibilities. Second, body fluid status should be elevated to distinguish the low sodium (low aldosterone action) and fast chloride types (normal aldosterone action). Finally, renin, aldosterone, and cortisol levels provide additional information regarding low aldosterone production and resistance to aldosterone action.

## Conclusion

Autoimmune-related adrenal insufficiency should be considered in the differential diagnosis of patients with MG who present with mild hyperkalemia. Early diagnosis of adrenal insufficiency can lead to timely steroid therapy.

## Data Availability

Not applicable.

## Abbreviations

ECF: extracellular fluid
MG: Myasthenia gravis (MG)
PAS: Polyglandular autoimmune syndrome
TTKG: Transtubular potassium gradient

## References

[1] Silvestri NJ, Wolfe GI (2012). Myasthenia gravis. Semin Neurol.

[2] Blanco Hernandez T (2009). Seronegative myasthenia gravis associated with other autoimmune diseases. Neurologia.

[3] Thorlacius S (1989). Associated disorders in myasthenia gravis: autoimmune diseases and their relation to thymectomy. Acta Neurol Scand.

[4] Dumas P (1985). Myasthenia gravis associated with adrenocortical insufficiency. Report of two cases. J Neurol.

[5] Kane CA, Weed L (1950). Myasthenia gravis associated with adrenocortical insufficiency; report of a case with post-mortem findings and a review of the literature. N Engl J Med.

[6] Okada T (1994). Myasthenia gravis associated with Addison's disease. Intern Med.

[7] Neary N, Nieman L (2010). Adrenal insufficiency: etiology, diagnosis and treatment. Curr Opin Endocrinol Diabetes Obes.

[8] Zhang B (2012). Autoantibodies to lipoprotein-related protein 4 in patients with double-seronegative myasthenia gravis. Arch Neurol.

[9] Gilhus NE (2009). Autoimmune myasthenia gravis. Expert Rev Neurother.

[10] Nacu A (2015). Complicating autoimmune diseases in myasthenia gravis: a review. Autoimmunity.

[11] Fang F (2015). The autoimmune spectrum of myasthenia gravis: a Swedish population-based study. J Intern Med.

[12] Fierabracci Alessandra (2016). Type 1 Diabetes in Autoimmune Polyendocrinopathy-Candidiasis-Ectodermal Dystrophy Syndrome (APECED): A “Rare” Manifestation in a “Rare” Disease. International Journal of Molecular Sciences.

[13] Betterle C (1996). Type 2 polyglandular autoimmune disease (Schmidt's syndrome). J Pediatr Endocrinol Metab.

[14] Bosch EP, Reith PE, Granner DK (1977). Myasthenia gravis and Schmidt syndrome. Neurology.

[15] McAlpine JK, Thomson JE (1988). Myasthenia gravis and Schmidt syndrome. Postgrad Med J.

[16] Konno S (2009). Autoimmune polyglandular syndrome type 2 with myasthenia gravis crisis. Neurologist.

[17] Seker M (2009). Myasthenia gravis and autoimmune Addison disease in a patient with thymoma. Clin Lung Cancer.

[18] Eleftheriadis T (2012). Differential diagnosis of hyperkalemia: an update to a complex problem. Hippokratia.

[19] Viera AJ, Wouk N (2015). Potassium Disorders: Hypokalemia and Hyperkalemia. Am Fam Physician.

[20] Lehnhardt A, Kemper MJ (2011). Pathogenesis, diagnosis and management of hyperkalemia. Pediatr Nephrol.

